# Are the expected benefits of requirements reuse hampered by distance? An experiment

**DOI:** 10.1186/s40064-016-3782-0

**Published:** 2016-12-20

**Authors:** Juan M. Carrillo de Gea, Joaquín Nicolás, José L. Fernández-Alemán, Ambrosio Toval, Ali Idri

**Affiliations:** 1 Department of Computer Science and Systems, University of Murcia, Murcia, Spain; 2 ENSIAS, Mohammed V University, Rabat, Morocco

**Keywords:** Experiment, Global software development, Internationalisation, Requirements engineering, Reuse

## Abstract

**Background:**

Software development processes are often performed by distributed teams which may be separated by great distances. Global software development (GSD) has undergone a significant growth in recent years. The challenges concerning GSD are especially relevant to requirements engineering (RE). Stakeholders need to share a common ground, but there are many difficulties as regards the potentially variable interpretation of the requirements in different contexts. We posit that the application of requirements reuse techniques could alleviate this problem through the diminution of the number of requirements open to misinterpretation.

**Results:**

This paper presents a reuse-based approach with which to address RE in GSD, with special emphasis on specification techniques, namely parameterised requirements and traceability relationships. An experiment was carried out with the participation of 29 university students enrolled on a Computer Science and Engineering course. Two main scenarios that represented co-localisation and distribution in software development were portrayed by participants from Spain and Morocco. The global teams achieved a slightly better performance than the co-located teams as regards *effectiveness*, which could be a result of the worse *productivity* of the global teams in comparison to the co-located teams. Subjective perceptions were generally more positive in the case of the distributed teams (*difficulty*, *speed* and *understanding*), with the exception of *quality*.

**Conclusions:**

A theoretical model has been proposed as an evaluation framework with which to analyse, from the point of view of the factor of distance, the effect of requirements specification techniques on a set of performance and perception-based variables. The experiment utilised a new internationalisation requirements catalogue. None of the differences found between co-located and distributed teams were significant according to the outcome of our statistical tests. The well-known benefits of requirements reuse in traditional co-located projects could, therefore, also be expected in GSD projects.

## Background

Distributed software projects have become a growing trend in recent years (Lima Peixoto et al. 2010; Colomo-Palacios et al. 2014). Furthermore, global software development (GSD) projects involve a heterogeneous mixture of cultures, geographical locations and time zones that require new approaches from different areas of knowledge if they are to be successful in this challenging and potentially rewarding environment (Damian et al. 2003). Multidisciplinary research is thus essential as regards increasing our knowledge of collective, cooperative and distributed work and how it should be applied in this context (Hinds and Kiesler 2002).

GSD provides organisations with significant benefits: lower staff costs owing to wage labour differentials between countries, a greater availability of skilled professionals who are not accessible in the immediate environment, better access to important markets or customers, longer working timetables when teams do not share the same time zone, and the enriched diversity the distributed participants’ experiences, techniques and skills. However, although working in a global environment has its advantages, there are also a number of drawbacks to consider (Noll et al. 2010; Holtkamp et al. 2015).

There are many obstacles to overcome in GSD: knowledge management, configuration management, cultural differences, communication, etc. GSD challenges traditional software engineering (SE) techniques (Ebert et al. 2001), thus affecting all the activities in the software development process, and requirements engineering (RE) in particular. Researchers within the SE community have, for various decades, recognised that RE is a complex process (van Lamsweerde 2000). The existence of poor requirements is acknowledged to be a major trigger of software project failure (Glass 1998; Cerpa and Verner 2009). Moreover, Cheng and Atlee (2007) emphasised the importance of drawing attention to GSD in the context of RE research. For these authors, it is essential to overcome the difficulties that GSD implies if RE is to achieve the efficient distributed development of software products.

A process model that can be specifically tailored to the needs of a given software development project should be founded on best practices in order to contribute towards supporting GSD (Ebert and Neve 2001). Numerous papers that study the risks of developing RE activities in a global environment have been published in recent years (Verner et al. 2014). New methods, techniques and tools are needed to find possible solutions to the main challenges related to RE in GSD (Ebling et al. 2009) (e.g. misunderstandings and misconceptions regarding the true meaning of requirements, owing to linguistic and cultural differences; barriers to the communication of stakeholders’ needs and expectations as regards the system to develop, owing to the distance between distributed teams).

The potential to reduce quality costs is growing significantly with the reuse of software units (Galinac Grbac et al. 2014). Many types of software units are commonly obtained from the various activities in the software lifecycle—e.g. design models, code, test plans, which can potentially become reusable assets. According to McClure (1997), there are two main reuse practices: “building for reuse” and “building with reuse”. In the first case, reusable knowledge units are initially identified, their key characteristics are extracted and represented through an abstraction process, and they are stored in a knowledge base for later use. In the second case the reusable knowledge units are sought from within the knowledge base in which they are stored, modified if needed, and combined with new knowledge to meet the needs arising from the current project.

The reuse of products of the RE process have, to date, been less frequently analysed than the reuse of design or code artifacts. It has been found, however, that the higher the abstraction level is, the greater the reusability benefits are Cybulski and Reed (2000) and Sommerville (2004). According to Rine and Nada (2000), requirements reuse provides the greatest improvement as regards productivity, quality and time-to-market in between the different software reuse activities that can be performed throughout the software development life cycle. Those findings have been corroborated by recent case studies conducted by other researchers (Pacheco et al. 2015, 2016; Goldin and Berry 2015). Many authors emphasise that the reuse of pre-existing sets of requirements identified during previous projects or from other domains can improve quality and reduce time-to-market (Robertson and Robertson 2006; Cheng and Atlee 2007). Software development companies should adopt requirements reuse as a key activity when their RE processes tend to take place in similar realms (Franch 2013). Nonetheless, very little work has been done to date with regard to the empirical study of requirements reuse in GSD.

We postulate that reusing requirements in GSD is at least as beneficial as doing so in traditional, co-located development, and that the added difficulties implied in GSD do not particularly affect this RE reuse strategy. Our manuscript is a contribution to the evaluation of catalogue-based requirements reuse in GSD through an experiment carried out with university students who were located in Rabat, Morocco (Mohammed V University) and Murcia, Spain (University of Murcia). They were therefore distributed in a nearshore scenario (Carmel and Abbott 2007). The experiment was carried out using an internationalisation (i18n) requirements catalogue (Toval 2011; Cos et al. 2012).

Our earlier work on this topic dealt with the comparison of non-reuse and reuse strategies in a distributed context (Carrillo de Gea et al. 2016; Fernández Alemán et al. 2016). The experimental data analysed in this manuscript were collected and used in the mentioned publications. In this work, however, the focus is on the assessment of the reuse approach in relation to the factor of distance (or proximity). Specifically, we aim to investigate whether the application of a set of catalogue-based requirements reuse techniques is affected by the proximity or distance of team members. To that end, we carry out our study using an appropriate data analysis method to check our assumption.

This paper analyses requirements reuse in GSD with the particular intention of shedding light on the possible benefits of two requirements specification techniques: parameterised requirements and traceability relationships. These principles are presented in the PANGEA (*Process for globAl requiremeNts enGinEering and quAlity*) framework (Carrillo de Gea et al. 2013), our reuse-based approach for RE in GSD. According to Cheng and Atlee (2007), two of the most pressing needs in RE at the present are GSD and requirements reuse. To the best of our knowledge, however, no other RE proposals currently tackle catalogue-based requirements reuse and GSD together. It was for this reason that PANGEA, a reuse-based RE method that explicitly addresses GSD, was devised. It revolves around a shared, centralised repository of reusable catalogues so as to foster requirements reuse, and integrates and builds on previous research (Toval et al. 2002a, b, 2008; Nicolás et al. 2009; Martínez et al. 2010).

The remainder of this article is organised as follows: “Methods” section presents the aim, hypotheses, techniques and study methods. “Results” section reports on the outcome of the statistical analysis. “Discussion” section provides a detailed discussion and appraisal of the study findings in relation to the key goals of this experiment, including the threats to the validity of this research. “Related work” section summarises previous work in the intersection of requirements reuse, experimentation and GSD. Finally, “Conclusions” section includes the conclusions and future work.

## Methods

### Aim

The main goal of our experiment is to study the effects of distance on the activity of requirements specification. In particular, the experiment is focused on a “building with reuse” process, since the identification of the reusable assets—i18n requirements in this case—had already taken place and the participants focused solely on the utilisation of these artefacts and the application of the reuse techniques (see “Requirements reuse techniques” section). We postulate that it is beneficial to reuse requirements in GSD, as occurs in traditional, co-located development (Rine and Nada 2000; Vavassori and Silva 2013). The approach used to study this issue concerns:

1. Considering a suitable GSD context in which requirements reuse may play a role. Our scenario involves people located in Murcia (Spain) and Rabat (Morocco), i.e. nearshore development:
  Temporal distance. The nodes are not in the same time zone, and the time difference between locations ranges from 1 to 2 h.
  Physical distance. The sites are separated by approximately 700 km.
  Socio-cultural distance. There are pronounced differences between the locations as regards language and culture.
  There are no large temporal and geographical distances involved in this scenario, but the ability to collaborate is, nevertheless, greatly affected by relatively small physical distances (Herbsleb 2007). According to Allen (1977), a strong anti-correlation exists between geographical distance and communication frequency between nodes, and a drastic decline in spontaneous communication and collaboration between individuals occurs when distance exceeds a threshold of around 50 m. In addition, the socio-cultural distance between Spain and Morocco is of great importance owing to considerable differences with regard to language, ethnicity, religion, customs and traditions. These *national* cultural differences account for the dissimilar classification of both countries according to a variety of cultural factors or dimensions, such as the indicators proposed by Hofstede et al. (2010), Hall (1959, 1976), and Hampden-Turner and Trompenaars (1993).
2. Determining techniques which might favour successful requirements reuse in GSD. In this case, parameterised requirements and traceability relationships.
3. Carrying out a controlled experiment focused on the study of these techniques in the proposed setting. More details on this matter are presented below.

The research goals have been outlined using the Goal/Question/Metric (GQM) framework (Basili and Rombach 1988). The GQM template (Basili et al. 1999; Wohlin et al. 2000) of the experiment is shown in Table 1.

**Table 1:** GQM template

Goal	The goal is to empirically analyse the application of catalogue-based requirements reuse techniques to improve requirements specification in GSD environments
Question	Is the application of catalogue-based requirements reuse techniques affected by the proximity or distance of team members?
Metric	Effectiveness (accuracy of the result), productivity (ratio of outputs to inputs) and perceived difficulty (ease of use), speed (rate at which the techniques are applied), quality (relative standard of the result) and understanding (comprehension of the techniques)
Goal definition template	
Object of study	The objects under study are requirements reuse techniques
Purpose	The purpose is to evaluate requirements specification techniques and team configurations, specifically as regards differences between co-located and distributed development
Quality focus	The quality focus is the effectiveness, productivity and subjective perception of requirements reuse techniques
Perspective	The perspective is the researcher’s point of view
Context	The study is run using university students as subjects based on a defined assignment with a reusable i18n requirements catalogue. The study is conducted as a blocked subject-object study

### Hypotheses

The following hypotheses were investigated:

H$$1_{0}$$:
*The application of catalogue-based requirements reuse techniques is not affected by the distance of team members in terms of:*

- H1.$$1_{0}$$: *Effectiveness*.
- H1.$$2_{0}$$: *Productivity*.
- H1.$$3_{0}$$: *Difficulty*.
- H1.$$4_{0}$$: *Speed*.
- H1.$$5_{0}$$: *Quality*.
- H1.$$6_{0}$$: *Understanding*.

These hypotheses are tested against the following alternative hypotheses:

H$$1_{A}$$:
*The application of catalogue-based requirements reuse techniques is affected by the distance of team members in terms of:*

- H1.$$1_{A}$$: *Effectiveness*.
- H1.$$2_{A}$$: *Productivity*.
- H1.$$3_{A}$$: *Difficulty*.
- H1.$$4_{A}$$: *Speed*.
- H1.$$5_{A}$$: *Quality*.
- H1.$$6_{A}$$: *Understanding*.

### Requirements reuse techniques

A reusable requirements catalogue that belongs to the i18n domain was utilised in this study. i18n is commonly defined as the activities undertaken to design software programs with a focus on their adaptation for use in different locations without the need for considerable modifications (He et al. 2002). It is not currently possible to obtain reliable information on software i18n from a single standard or regulation; instead, there are more than 20 documents that encompass information on this topic. These disparate sources were therefore studied in order to gather i18n knowledge in a catalogue of reusable requirements (Toval 2011; Cos et al. 2012) called I-CAT (*Internationalisation CATalog*).

The most important techniques used by the participants in the experiment, namely parameterised requirements and traceability relationships, are not specific to the PANGEA framework, but are rather of general interest and applicability in RE. In addition, in order to complement facts or details about requirements, attributes are used to accompany these requirements as meta-information. There are different types of requirements, but a set of generally pertinent attributes can be associated with all of them. Exemplifying, *UniqueIdentifier* (compulsory) is a code that helps identify each requirement in relation to other project requirements (e.g. SRS1, SRS2, SRS3 and so on); *Text* (compulsory) is a sentence written in natural language (NL) that documents the requirement; and *Priority* is a value that represents the order of precedence of the development assigned to the requirement (e.g. *High*, *Medium* and *Low*).

Some examples of the kind of requirements and traceability relationships included in the i18n catalogue and extensively applied during the experiment are shown below. We also use the actual *UniqueIdentifier* of each requirement in order to identify it unambiguously in the catalogue.

At the level of requirements, and leaving aside formal approaches such as OBJ3 (Goguen and Winkler 1988), parameterisation has been dealt with in the form of: (1) parameterised features such as FODA (Kang et al. 1990) and FORM (Kang et al. 1998); and especially (2) parameterised requirements (see e.g. Lam et al. 1997; Firesmith 2004). Parameterised requirements are particularly relevant for requirements reuse processes, since they allow requirements engineers to define variation points in the specification. This means that a number of possible values can be defined for the parameter, thus allowing the most appropriate alternative with which to shape the desired product to be selected at reuse time. For instance:

- SRS30. *The tool shall allow the display, or method of presentation of the content, to be transformed using [styling mechanisms]*.
  The square brackets serve to specify the parameter between them: *[styling mechanisms]*.
  In the event that SRS30 is selected for reuse, the instantiation of the above parameter with one of several alternative values takes place in reuse time:
  - *Cascading style sheets (CSS)*.
  - *System-based display settings*.
  - *XSL transformations (XSLT)*.
  - *Other*.

Ideally, requirements would not change throughout the software development life cycle, but unfortunately it has been acknowledged that this does not occur in real world projects (Robertson and Robertson 2013; Wiegers and Beatty 2013). Requirements are not static, and it is therefore necessary to manage changes made to them. As requirements change and the requirements specification evolves, other requirements may be affected. The creation of traceability relationships, or links, between requirements can help deal with this complex situation (Kuang et al. 2015). The same principles apply to the reusable requirements included in a catalogue. Carrillo de Gea et al. (2013) defined a very simple traceability model that was used in the experiment. It is summarised and illustrated with examples below:

- *Parent-child* This kind of relationship characterises a general higher-level requirement by means of a series of specific lower-level requirements. For instance:
  SRS5. *The tool shall provide the user with individualisation and adaption characteristics by means of user profiles (PNPs) or group profiles*.
  SRS5.1. *The tool shall allow the user to customise it to their personal needs (individualisation), adapting the contents and navigation according to the user group or role*.
  SRS5.2. *The tool shall monitor the user’s behaviour and adapt itself to the user’s goals, which should be inferred from the observed behaviour*.
  SRS5.3. *The tool shall recommend contents that are potentially relevant to the user, based on the behaviour of other users or a user group*.
  SRS5 *Parent-child* SRS5.1, SRS5.2, SRS5.3.
- *Requires* This relationship describes the reliance of a given requirement on ahother requirement. If R1 *Requires* R2, then it is mandatory to reuse R2 whenever R1 is reused. For instance:
  SRS4. *The tool shall allow the user to create a personal needs and preferences (PNP) description by means of [PNP description]*.
  SRS10. *Once a person has a PNP, (s)he should be able to completely remove his/her PNP statement as needed*.
  SRS4 *Requires* SRS10.
- *RelatedTo* This type of relationship expresses the reliance of two requirements on one another. If R1 is *RelatedTo* R2, and R1 is reused, then the possible reuse of R2 should be taken into account, since R2 refines or supplements R1. The reuse of R2 is not mandatory in this case. For example:
  SRS11. *The tool shall allow the user to create multiple PNPs in order to have a convenient way to switch between several sets of needs and preferences for different situations*.
  SRS12. *Once a person has a PNP, (s)he should be able to move his/her PNPs to other systems for reuse*.
  SRS11 *RelatedTo* SRS12.

### Data collection method

The participants were Computer Science and Engineering students in either their last years at university or their first years of postgraduate studies with similar training and experience in SE and RE. Furthermore, they were taught the foundations of the techniques to apply during the activity to ensure that they had a common level of knowledge of the topic. Those taking part in the experiment were therefore familiarised with both RE and requirements reuse. A further requirement for participation was a good command of technical English in order to be able to follow the instructions during the experiment. We ensured that all the participants were informed of the procedure, and that their choice as to whether or not to participate was voluntary by requesting written consent for participation.

The task defined in the experiment was carried out by teams of two people. We created three participation modalities depending of the composition of the team: (1) global (GLO), one student from the University of Murcia (UMU) was paired up with one student from the Mohammed V University (UMV); (2) co-located in Murcia (CLM), two students from the UMU were paired up; and (3) co-located in Rabat (CLR), two students from the UMV were paired up. In general, the working language of the experiment was English. Informal communication in the native language was only allowed in the case of participants in the co-located modalities, thus simulating a real software development context in which teammates on the same site usually share the same language. Nevertheless, work deliverables had to be presented in English, in both the global and co-located modalities.

A total of 31 students initially participated in the activity. Of the 31 participants, 19 were male and 12 were female. In terms of percentages, this signifies that males comprised 61.3% and females 38.7%. The mean age of the sample was within the range of 21–25. There were 15 participants from the UMV and 16 participants from the UMU. A total of 14 teams of two students and one team of three students were defined so as to be able to place all the students in teams. Seven teams encompassing 15 students were assigned to the co-located modality (either CLM or CLR), and they were the control group. Eight teams encompassing 16 students were assigned to the global modality (GLO), and they represented the experimental group in the experiment.

The following measures were taken at the beginning of the study: (1) the participants were taught the foundations of the techniques to be applied during the experiment; (2) the procedure of the experiment was explained to the participants; and (3) all the necessary documentation was handed out. The duration of the experiment was two weeks, starting on November 26th, 2012. In fact, the estimated time required to finish the proposed task was in the order of hours, but the initial contact, communication, meetings and negotiation overheads also had to be included in the two week period. Moreover, an additional requirement was defined for the co-located teams (either CLM or CLR), which was that they had to hold at least one face-to-face meeting.

Detailed information on the experimental task is supplied in “Appendix”. The task to be completed by the participants was concerned with the elicitation of a set of requirements from a requirements catalogue. Specifically, the participants had to search for and reuse requirements related to a specific software i18n topic using the i18n requirements catalogue and the requirements specification techniques outlined in “Requirements reuse techniques” section. The solutions of the task submitted by the participants were the input used to measure *effectiveness*. The participants also filled in a questionnaire (see “Appendix”) in which they reported the time spent on the experimental task, which was needed to calculate *productivity*. Their perceptions of the techniques and results were also gathered together by means of the questionnaire and were used to measure *difficulty*, *speed*, *quality* and *understanding*. A detailed description of all these indicators is provided in “Data analysis method” section. Furthermore, the questionnaire design was based on similar studies reported in literature (Moros et al. 2013).

### Data analysis method

**Fig. 1:**
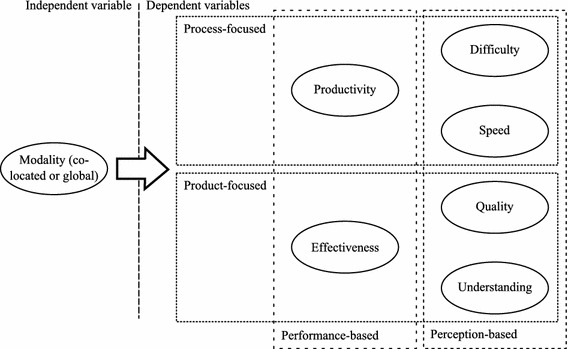
Theoretical evaluation model

We defined a set of six variables in this study, and a Likert scale was used in five of them: *effectiveness*, *productivity*, *difficulty*, *speed*, *quality* and *understanding*. Our variables can be classified into either *performance-based* variables or *perception-based* variables (Abrahão et al. 2011), as shown in Fig. 1. The diagram illustrates the theoretical model that was defined in this experiment, which was adapted from the Method Evaluation Model (Moody 2003) so as to fit in the assessment of requirements specification techniques. The mentioned variables were therefore chosen for measurement because of their potential to precisely characterise the application process of the requirements specification techniques under study.

The *effectiveness* represents the accuracy of the result and can be measured by evaluating the artefacts that are produced by using the requirements specification technique (Abrahão et al. 2011). Improving systematic reuse is critical as regards increasing the software teams’ *productivity* (Altintas et al. 2012). The *difficulty*, or effort required to learn and use a technique, can be described as the perceived “ease of use” or the degree to which an innovation is perceived as difficult to understand and use (Krzanik and Similä 1997). The *speed* is perhaps the single most important success factor in modern high technology businesses, especially in a GSD environment (Herbsleb et al. 2001; Herbsleb and Mockus 2003). Effective coordination within a GSD project requires planning and agreeing on achievable milestones; effective monitoring will help oversee ongoing progress as regards *quality*, among other factors (Richardson et al. 2012). The *understanding* of requirements is essential for their validation among stakeholders (Abrahão et al. 2011); there are empirical studies which suggest that problem-solving tasks can be used as an instrument with which to measure understanding (Bodart et al. 2001).

*Effectiveness* was objectively assessed by the researchers on the basis of the correctness of the outcome of the task execution (1-*Very good*, 2-*Good*, 3-*Fair*, 4-*Poor*). *Productivity*, often defined as output divided by the effort required to produce that output (Maxwell and Forselius 2000), was measured in *requirements per hour* (Seyff et al. 2009), which is the amount of requirements reused per hour per team (i.e. $Productivity = \frac{requirements}{hour}$). The other variables reflect the participants’ subjective perceptions and were obtained using a five-point Likert scale. In this respect, *difficulty* measures how hard the techniques are (1-*Easy*, 5-*Difficult*), *speed* measures the agility of the techniques (1-*Fast*, 5-*Slow*), *quality* measures the quality of the requirements obtained (1-*High*, 5-*Low*), and *understanding* measures the comprehension of the requirements obtained (1-*Good*, 5-*Bad*).

According to the description provided above, *productivity*, *difficulty* and *speed* deal with aspects of the *process*, and *effectiveness*, *quality* and *understanding* tackle the *product*. The variables in the study are thus not isolated, and this fact is graphically reflected in the dotted lines around the sets of variables in Fig. 1.

Depending on the specific characteristics of the data collected, parametric or non-parametric tests were carried out (Burns and Burns 2008), to establish in which classifying variables there are significant differences between team configurations. In other words, hypothesis tests were applied so as to support our results empirically. Statistical hypothesis testing provides a means of formally checking whether or not the diversity of indicators and scores achieved by the teams belonging to each modality is definitely noteworthy. Practical guidelines for statistical tests in SE were followed (Arcuri and Briand 2011).

Parametric tests require certain assumptions made about the data to be true, specifically the variables must have a normal distribution. To that end, the Shapiro–Wilk (*W*-statistic) formal statistical test was used. In the case that one variable does not have a normal distribution, a non-parametric test must be applied. Two-independent-samples *T* tests (*T*-statistic) or Mann–Whitney *U* tests (*U*-statistic) were therefore used—i.e. parametric and non-parametric tests, respectively, depending on the result of the *W* test.

Some common statistical procedures, such as the two-independent-samples *T* tests, assume that variances in the populations from which different samples are drawn are equal. The null hypothesis that the population variances are equal, also known as the *homogeneity of variances* assumption, was assessed by means of Levene’s test (*F*-statistic) before applying the *T* tests. Levene’s test does not require normality of the underlying data. When Levene’s test is significant, modified procedures that do not assume equality of variances are used.

## Results

At the end of the recruiting process, a participation rate of 93.55% was obtained (29 out of 31 individuals took part in the study). One of the CLR teams, formed of two students, was discarded when it was identified as an outlier. What is done with identified outliers depends largely on why they are in the data (Osborne and Overbay 2008). The participants are responsible for the correctness of the data with which the experimenters are provided, and the outlier should therefore be removed from the dataset if that data point is judged to be there illegitimately (Barnett and Lewis 1994; Osborne and Overbay 2008).

**Fig. 2:**
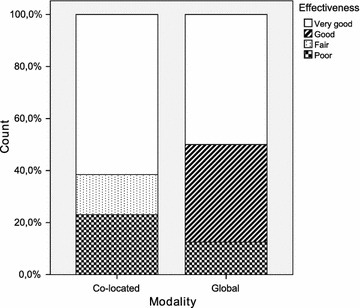
Effectiveness (1-*Very good*, 4-*Poor*)

**Fig. 3:**
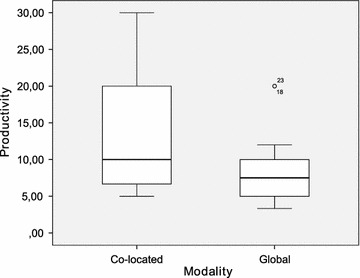
Productivity (requirements per hour per team)

**Fig. 4:**
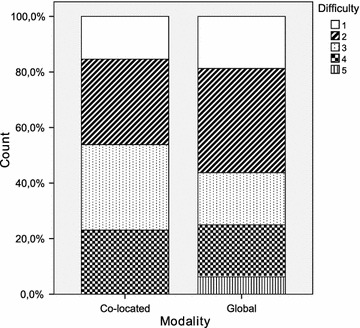
Difficulty (1-Easy, 5-Difficult)

**Fig. 5:**
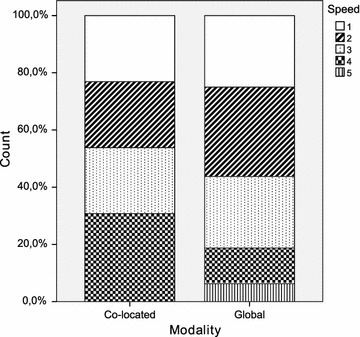
Speed (1-Quick, 5-Slow)

**Fig. 6:**
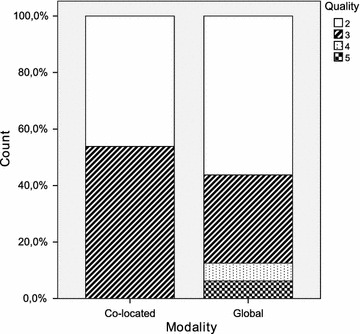
Quality (1-High, 5-Low)

**Fig. 7:**
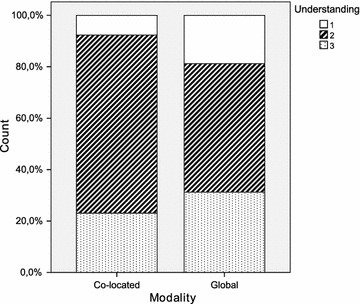
Understanding (1-Good, 5-Bad)

Table 2 provides a summary of the principal statistics computed from the dataset. It includes the arithmetic mean and median, both of which are measures of central tendency. The standard deviation, minimum and maximum are additionally shown as measures of dispersion. Furthermore, a set of diagrams is presented in Figs. 2, 3, 4, 5, 6 and 7, which depict two kinds of graphs, namely: stacked bar graphs and boxplots (Ott and Longnecker 2010).

**Table 2:** Descriptive statistics

Modality	Co-located ($\hbox {N}=13$)			Global ($\hbox {N}=16$)		
Statistic	Mean	Median	SD	Mean	Median	SD
Effectiveness	2.00	1.0	1.35	1.75	1.5	1.00
Productivity	13.14	10.0	7.91	8.82	7.5	4.95
Difficulty	2.62	3.0	1.04	2.56	2.0	1.21
Speed	2.62	3.0	1.19	2.44	2.0	1.21
Quality	2.54	3.0	.52	2.62	2.0	.89
Understanding	2.15	2.0	.55	2.12	2.0	.72

*SD* std. deviation

To summarise the descriptive information presented above, the global teams achieved a slightly better performance than the co-located teams as regards *effectiveness*. The reason for this could be that the *productivity* of the global teams was worse than that of the co-located teams. In this respect, the amount of time and effort spent on the RE activities is a determining factor of the final outcome (Hofmann and Lehner 2001; Hooks and Farry 2001), which we have quantified in this study using the *effectiveness* measure shown previously.

It would appear that the global teams have a better perception of the experimental task than the co-located teams. This is true for all the subjective measures with the only exception of *quality*. It is worth noting, however, that the magnitude of the differences is very small throughout all the variables. With regard to the dispersion of the measures, there is more variability in *difficulty* and *speed*, and less variability in *quality* and *understanding*. This occurs in the cases of both the co-located teams and the global teams.

Statistical inference techniques were also applied in order to study the differences between the co-located and the distributed teams. The results of the *W* test are shown in Table 3. This test suggests that the hypothesis of normality should be rejected if p value $\le$.05. In these cases, we can accept the alternative hypothesis and conclude that the samples are not taken from a normal distribution.

**Table 3:** Results of the normality test

Variable	Modality			
	Co-located		Global	
	Statistic	p value	Statistic	p value
Effectiveness	$W = .688$	*.000*	$W = .715$	*.000*
Productivity	$W = .850$	*.028*	$W = .806$	*.003*
Difficulty	$W = .896$	.116	$W = .906$	.100
Speed	$W = .861$	*.039*	$W = .906$	.100
Quality	$W = .646$	*.000*	$W = .729$	*.000*
Understanding	$W = .733$	*.001*	$W = .814$	*.004*

Two-independent-samples *T* tests (*T*-statistic) and Mann–Whitney *U* tests (*U*-statistic) were conducted so as to compare requirements reuse in co-located and distributed conditions using the performance and perception-based variables included in our theoretical evaluation model (see Fig. 1). Table 4 provides a summary of the statistical tests that have been performed in this work. Note that 5 out of 6 tests were *U* tests, whereas the remaining one was a *T* test.

**Table 4:** Hypothesis testing: two-independent-samples *T* test and Mann–Whitney *U* test

Variable	Statistic	p value
Effectiveness	$U = 103.0$, $Z = -.048$	.961
Productivity	$U = 71.0$, $Z = -1.470$	.142
Difficulty	$T(27) = .124$	.902
Speed	$U = 93.5$, $Z = -.474$	.635
Quality	$U = 100.5$, $Z = -.173$	.863
Understanding	$U = 103.0$, $Z = -.050$	.960

Significance level: .05

There were no significant differences—a result is said to be statistically significant if it is unlikely to have occurred by chance—between the scores for co-located and distributed conditions. The null hypothesis H$1_0$ cannot, therefore, be rejected at the .05 level of significance. In other words, our findings support the idea that the treatment, or distribution modality, does not lead to a statistically significant variation in the variables of the study. These results suggest that the factor of distance (or proximity) does not affect catalogue-based requirements reuse. In the particular case of *productivity* (H1.$2_0$), although there were no significant differences ($U = 71.0$, $Z = -1.470$, $p = .142$) between the scores for co-located ($M = 13.14$, $SD = 7.91$) and global ($M = 8.82$, $SD = 4.95$) conditions, the result is noticeable since the p value is relatively close to .05.

## Discussion

When the appropriate conditions exist for a requirements reuse approach to be applied, its advantages over developing a software product from scratch are well documented in literature for traditional, co-located settings (Rine and Nada 2000; Pacheco et al. 2015; Vavassori and Silva 2013). The key finding of our study is the absence of significant differences between teams, regardless of proximity or distance, with regard to the *effectiveness*, *productivity*, *difficulty*, *speed*, *quality* and *understanding* of catalogue-based requirements reuse. Our results therefore suggest that the requirements reuse techniques presented in this work can help co-located and distributed teams achieve similar performance in the requirements specification activity in software development projects.

The techniques described in this paper are focused on NL requirements, a prevalent form of requirements specification that is widely used in industry (Yang et al. 2010; Mavin 2012; Ott 2013). Textual specifications are useful to express and communicate requirements in a language that both stakeholders and developers can understand. However, this approach is prone to certain problems because NL is inherently ambiguous, which could lead to different interpretations depending on the particular context (Mavin 2012; Mavin et al. 2009; Mavin and Wilkinson 2010). Specifically, when stakeholders who are globally distributed work together, these problems may appear and jeopardise the success of the project. There may consequently be difficulties when understanding the requirements and exchanging the knowledge that they specify about the product. The implementation of a strategy that allows the reuse of requirements that are specified in NL in GSD environments, which includes the reuse techniques presented in “Requirements reuse techniques” section, may help to overcome those pitfalls. We believe that the results of this experiment suggest that requirements reuse can contribute to remove the line that separates co-located and distributed RE.

A straightforward reuse-based RE process has many advantages, since if the method is sufficiently simple, it can be easily implemented by companies and improve their working practices as regards RE (Carrillo de Gea et al. 2013). Reuse activities require sufficient specific training to obtain satisfactory results, and all previous knowledge or experience related to software development in general cannot be considered a substitute (Cybulski 1996). We therefore believe that the simpler the method, the less training time is required. Our experiment confirms that the requirements reuse techniques presented can be effectively applied by both co-located ($M = 2.00$, $SD = 1.35$) and global ($M = 1.75$, $SD = 1.00$) teams, with a very small training effort.

The participants in this study applied the requirements reuse techniques in a manual fashion. This implies that they had to look up the reusable requirements in the catalogue, search for the traceability relationships between requirements in the traceability matrix, discover the set of related requirements and instantiate the requirements’ parameters where appropriate. They had to undertake these activities without the aid of specific and integrated tool support. Nevertheless, both the control and experimental groups carried out the task under the same conditions, signifying that although the availability of automated tool support would have sped up the application of the requirements reuse techniques (Toval et al. 2008), it is to be expected that both participation modalities would have undergone the same impact and their results would have improved to the same degree. The key finding of this work, described above, would not therefore have been essentially modified by the availability of automated tool support.

### Limitations

The threats to the validity (Wohlin et al. 2000) of the study are presented below.

#### Conclusion validity

The assumptions of the statistical tests were followed when analysing the results. In this respect, when data normality could not be assumed, we performed the statistical analysis using a non-parametric test (i.e. Mann–Whitney *U* test) rather than a parametric test (i.e. a two-independent-samples *T* test). We believe that the most important limitation found in this research is concerned with the relatively small size of the sample. Nevertheless, the studies reported in the related literature (see “Related work” section) usually involve a similar amount of participants.

Another potential threat in this study is the low reliability of the measures. Of the factors that may have led to this problem, we can mention poor question wording and bad instrument design. We have specifically addressed both factors in order to mitigate this threat.

#### Internal validity

Some of the t in this experiment may be owing to confounding factors that can be categorised into individual- and subject-related parameters (Siegmund and Schumann 2015). Individual causes (e.g. fatigue, motivation, personality) were mitigated by configuring the teams of students in a random fashion within each modality (i.e. GLO, CLM, CLR). Moreover, the fatigue effect was limited by giving the participants ample time to perform the tasks. Students’ motivation was generally high, since the activity was included in one of the units of which their course is formed. Threats from other potential confounding factors (i.e. familiarity with RE, training level) were mitigated during the initial phases of the study. The demographic analysis showed that the participants had similar experience in RE. Furthermore, they all received specific training in order to normalise their knowledge.

With regard to subject-related parameters, evaluation apprehension caused by the fear of being evaluated may bias participants’ responses towards what they perceive to be better. We avoided this influence by assuring the participants that they would not be evaluated on the basis of their opinions, and encouraged them to answer honestly. Moreover, in relation to evaluation apprehension, the Hawthorne effect could make the participants behave differently when observed (Gillespie 1993). This threat was mitigated by not revealing the hypotheses of the study to the participants.

#### Construct validity

The instrumentation might have influenced the construct validity of the experiment. The study task was not standard, given that we were not aware of similar works. We attempted to include the most relevant artefacts and techniques involved in a catalogue-based requirements reuse process.

Another instrument, the questionnaire, was carefully developed by consulting literature and experts. This helped identify possible sources of potential problems and their subsequent prevention or solution. Furthermore, the design of the questionnaire was based on standard approaches and scales (Oppenheim 2000). Test–retest reliability for stability and reliability testing, measurement of internal consistency, use of a gold standard to improve criterion validity, factor analysis to test the construct validity of the questionnaire, and inter and intraobserver concordance analysis were not, however, carried out.

A further threat to construct validity is mono-method bias. Although we obtained various observations regarding each variable in the study by means of the questionnaire, only subjective impressions were collected in the case of some of the variables in the experiment. Since in these cases we collected the data using an unsupervised questionnaire survey, there was no means of guaranteeing that the participants had interpreted the questions in the same way as the researchers (Mäntylä and Lassenius 2006).

#### External validity

This experiment was carried out in an educational context. The activity was part of a course unit, and its magnitude and complexity were thus necessarily limited to the time available. It is not, therefore, possible to guarantee that the study objects represent the real world of software development projects. However, the study was planned, designed and executed to be as realistic and aligned with industry practices as possible.

External validity may also have been threatened by the fact that the participants were students. It is not, therefore, possible to ensure that our study participants are representative of real world software professionals. Nevertheless, scientific testing with students is considered viable as long as the tasks that are to be executed do not demand experience in industry (Basili et al. 1999; Svahnberg et al. 2008). Furthermore, it is still unclear whether there are actual differences between students and professionals in industry (Runeson 2003).

## Related work

In this section, previous work on experiments in RE education (see “Experiments in RE education” section) and GSD education (see “Experiments in GSD education” section) will be presented.

### Experiments in RE education

In a recent systematic mapping study on RE education (Ouhbi et al. 2015), 79 papers were selected and categorised according to five classification criteria: research type, empirical type, contribution type, RE activity, and curricula. Around half of the papers were case studies, surveys and experiments. Although these papers reported empirically validated experiences, a few papers carried out an appropriate statistical analysis (Arcuri and Briand 2011).

A games-based learning (GBL) application to teach requirements gathering and analysis at tertiary education level was proposed (Hainey et al. 2011). GBL was compared to traditional methods of SE education using five experiments. The combined experiment involved 92 students. The research performed indicated that a GBL approach may be more suitable at university level than college level when teaching requirements collection and analysis. The research also showed that this approach can be just as effective as role-playing and more effective than paper-based case studies.

A study of team projects of 4–5 students on an RE course compared the exam responses of the Spring 2005 students with those of the Spring 2006 term (Ludi 2007). A total of 17 students were involved. One group of two teams took part in projects using accessibility requirements while another group of two teams did so using moderate accessibility requirements. Students from the Spring 2005 term, with no external stakeholder interaction, demonstrated lower levels of accessibility understanding.

An experimental approach on teaching RE by using social simulations rather than software simulations was carried out on a system development course at Narvik University College (Danielsen 2010). The authors aimed to make the students learn through interactions with real people. The students were divided into two groups: one group without previous experience of software development or programming languages and another that was familiarised with both object orientation and programming. This study used 15 and 18 students each year. No statistical analysis was conducted.

A teaching model for security RE was validated in an SE management programme at University of Detroit Mercy (Mead et al. 2009). The Security QUAlity Requirements Engineering (SQUARE) method for secure software RE was used in two case studies with university students. Two groups of graduate students were defined: the experimental group used the SQUARE method, whereas the control group was given lectures on the topics of the IEEE Std. 830 (1998). Although no statistically significant differences were attained after analysing pre and post test results, the p value of .09 in the case of the treatment group indicated that the SQUARE treatment led to a noticeable rise in security-related knowledge. In the case of the treatment, the groups had between 12 and 15 members.

### Experiments in GSD education

A systematic literature review on the evidence in GSD-related research literature reported that the amount of empirical studies in GSD is relatively small (Šmite et al. 2010). It can be argued that there is an important amount of research work done in the field of Global Software Development Education (GSDE) (Monasor et al. 2010), but experiments are not yet common in spite of the need for this type of research work to validate theories in SE in general, and RE in particular, and draw solid conclusions backed by empirical evidence.

A total of 32 students experienced the iterative development of a requirements specification in GSD projects by playing the roles of client and developer in the context of an SE course (Damian et al. 2005, 2006). The course emphasised the learning of requirements management activities in frequent synchronous computer-mediated client-developer relationships and created an environment with significant temporal, geographical and sociocultural distance. This course was taught by means of the collaboration of three universities in disparate locations (Canada, Australia and Italy). The authors reported some of the challenges encountered and assessed the success of the course. However, no statistical analysis was carried out.

An educational model for software quality assurance through which students from three countries can work together with pedagogical value on all sides was presented in Gotel et al. (2008, 2009). Seven graduate students were included as internal mentors and external auditors to help ensure the quality of a GSD project involving 27 students from the three locations (Pace University, Institute of Technology of Cambodia and University of Delhi). Both process and tooling were addressed in the project. With regard to RE, the gathering, elicitation and validation of requirements were emphasised in order to focus further on quality. No specialised RE tool was used. No statistical analysis was conducted.

An offshore client/vendor relationship was replicated in the framework of a GSD educational initiative (Adya et al. 2007). The analysis and design were conducted by students at the Management Development Institute Gurgaon (MDI), whereas Marquette University (MU) teams only provided high level descriptions of projects. Detailed requirements were gathered by MDI teams through subsequent client interactions in a virtual mode. There were global (MU-MDI) and co-located (MDI-MDI) team interactions. A survey was conducted to measure the motivation, comfort and learning effectiveness of the participants using a 7-point Likert scale, followed by a quantitative assessment of the students’ perceptions.

Academic effectiveness, impact on SE learning and students’ perceptions of outsourcing were determined by means of an experiment on an advanced SE course (Honig and Prasad 2007). Teams made up of 4–5 students (with a total of 40 students) at two universities developed game playing programs and outsourced parts of their systems to the other university. Among other deliverables (e.g. project management plan, system design document), each team prepared a requirements analysis document. Descriptive statistical analysis results were reported, which were based on both the students’ self evaluations and assessments by faculty members. The results indicated that the course was effective, since the students changed their perception of outsourcing and realised the value of good communications.

A global project course involving two to four sites, both from Europe and the USA, and 30–40 students each year was taught for six years (Gloor et al. 2011). The students formed project teams spanning several sites, and jointly performed creative tasks, thus learning both the course contents and how to effectively work together in multicultural and multidisciplinary distributed teams. No statistical analysis was conducted in this study. Ten tips to succeed in GSD were followed on a student project performed as a part of a distributed SE course, which was organised by three universities (University of Zagreb, M?lardalen University and Politecnico di Milano), and designed as a combination of lectures, guest presentations and seven globally distributed projects (Crnković et al. 2012). The proposed tips were significant but not sufficient for a positive project outcome. The final result was determined by the overall composition of people and processes—supported by tools. No statistical analysis was carried out.

A method for teaching collaborative software development in realistic distributed multidisciplinary environments was applied by three Texan universities (Burnell et al. 2002). In the analysis activity, students created the requirements model, which included the specification of use cases to capture functional requirements. Downstream software development activities were also included in the process. No statistical analysis was reported. An organising framework with which to study multicultural distributed learning teams was proposed in Swigger et al. (2009). Learning team performance and its relations with culture, individual differences, and attitudes toward collaborative work were studied by starting from two GSD projects involving 152 students from the USA, Panama, UK and Turkey. Extensive statistical analysis was carried out. The findings showed that culture and attitudes about groups had the most effect on team performance, followed by individual characteristics.

An approach to combine and synchronise class teaching of SE with actual software development work in a GSD setting designed to simulate small companies was shown in Petkovic et al. (2006). This experience involved two universities (San Francisco State University and Fulda University), around 10 local groups (4–6 students) and 3–5 global groups (3 students from each university). Analysis of effectiveness based on several assessment methods—including student questionnaires—were conducted, although statistical results were provided to a limited extent. The GSD teaching methods used in a distributed project-oriented course jointly carried out by two universities from Sweden and Croatia were described in Bosnić et al. (2010). This course enabled students to be more engaged in real-world situations, by interacting with customers from industry, local or distributed customers in universities, distributed customers in SE contests or being involved in an ongoing project, thus simulating company merging. No statistical analysis was carried out, and only qualitative results were provided.

Students from Rowan University and Fairfield University collaborated in several real-world projects from an industrial organisation to be undertaken on a theoretical SE course (Rusu et al. 2009). A total of 20 students participated on the course. Teams of 3–4 students were created, depending on the number of projects available. Reflections on both the course outcome and the students’ perspective were reported, but no statistical analysis was provided. Students from the University of Limerick (UL) participated in two projects that provided them with a first-hand learning experience of working within GSD teams (Richardson et al. 2007). The first project ran for two years and was conducted in cooperation with Siemens so as to provide students with real-life GSD experience (Richardson et al. 2006). It involved seven distributed student teams from five universities in four countries. In each year, one team of five people from the UL participated. The second project was run with Ball State University, 12 students participated, and it was focused on virtual team software testing. The nature of the research was qualitative, and it was concluded that mimicking real work settings has educational benefits for problem-based learning environments.

## Conclusions

This work provides a new assessment of requirements reuse techniques in GSD by means of an experiment carried out with students. It is worth noting that Šmite et al. (2010) analysed the evidence in GSD-related research through a systematic literature review, and found that the amount of empirical studies in GSD is relatively small. On the other hand, the reuse of software is acknowledged to be an answer to the three main drivers of the software business, namely: *faster*, *better* and *cheaper*. Chernak (2012) conducted an industrial survey in which the benefits that the reuse of requirements can provide software companies with were confirmed to be coherent with those reported for the reuse of other types of software artefacts.

According to Ebert (2012), we could have expected GSD to demand up to 20% additional effort. There are a number of reasons for this (e.g. coordination and communication overheads, misunderstandings and misconceptions, lack of mutual trust). Contrary to expectations, our findings concerning the requirements reuse techniques did not reflect a decrease or deterioration in effectiveness, productivity or subjective perception in the case of global teams when compared to co-located teams. The results were similar in both team modalities. According to our experiment, we therefore conclude that the requirements reuse techniques will provide the same benefits in GSD as in traditional co-located software development projects.

In addition to the results presented in this work, in the same experiment we carried out an initial study of the applicability of other specification techniques, namely requirements templates and collaborative tagging. The results were encouraging and we plan to report these findings in future work. We also plan to replicate this experiment in the short term. In this respect, we shall include requirements templates and collaborative tagging as techniques in our reuse-based RE method and study their usefulness as regards promoting requirements reuse. The study will additionally include automated tool support for our reuse-based method. We thus expect to be able to validate our approach for RE in GSD as a whole (i.e. including both the method and the automated tool support).

Aspects related to the sustainability of the software development process have become increasingly important in recent times (Penzenstadler et al. 2012). In future work, we aim to devise more sustainable RE approaches, and the reuse-based requirements specification techniques put into practice in this work could be a promising contribution in this regard. There are many potential benefits of using requirements catalogues to promote the reuse of software artifacts. More requirements reuse means more productivity, which is normally pursued by companies, because it improves the business results. Another way of looking at this is the lower cost—i.e. time, effort, resources—at which software can be produced. As a result, companies could reduce the energy consumption and environmental impact associated with software development projects. For example, the experimental results presented in this work are supportive of the application of catalogue-based requirements reuse in GSD, which could encourage companies to resort to teleworking more often, thus reducing their carbon footprint.

## Appendix: Experiment details

The task statement is shown in Table 5. It includes the guidelines and information on the experiment that the participants were given at the beginning of the activity. The participants in the experiment had to fill out the questionnaire presented in Table 6 once the experimental task had been completed. *Q1* was used to obtain the time the participants spent on the proposed task. A typical five-point Likert scale (1-“Completely Agree”, 5-“Completely Disagree”) was used in *Q2–Q5*. *Q6* was used to collect the means of communication used by the participants in the experiment. *Q7* was used to gather optional feedback from the participants.

**Table 5:** Task statement

**Context** *Company1* has experience in selling one certain kind of products in its local market and wishes to improve its results by means of expanding its business to new customers. To that end, people in charge of *Company1* think that the better way to accomplish this objective is selling their products on the Internet. In addition, as part of their expansive strategy to other markets, the company wishes to encourage and favour sales out of the national market	
**Goal** The final goal of this task is to elicit software requirements related to software i18n, which can serve *Company1* in order to develop a website for selling products on the Internet. During the execution of this task, reuse-based RE techniques will be applied, namely parameterised requirements and traceability relationships	
**Task** The task will be performed in teams of two people. The team have to reuse requirements related to a specific topic of software i18n starting from a requirements i18n catalogue, already predefined. This reusable requirements catalogue is based on standards that contain i18n information and guidelines (e.g. ISO/IEC 24751, ISO 9241-151, W3C, and CWA 14928X). Traceability relationships between requirements and parameterised requirements will be used during this process	
**Provided materials** Participants will be provided the following materials	
1. Task statement (this document)	
2. Specific assignment of each team	
3. Generic i18n requirements catalogue in the form of an SRS	
4. Traceability matrix including the relationships between the requirements in the catalogue	
5. Summary of the foundations of the techniques to be applied	
6. Questionnaire	
**Planning** The stipulated period for the execution of this tasks is 2 weeks, starting from the date in which this tasks statement is handed in	
**Evaluation**. In order to proceed to the evaluation of the task, the teams and the team members individually when appropriate have to submit the task results and the questionnaire answers within the stipulated period. All outcome of the experiment must be written in English	
**Glossary**. Terms are listed in alphabetical order	
*Elicitation*: the task of identifying the various types of requirements from various sources including standards, project documentation, interviews, etc.	
*Parameterised requirement*: requirement that contains parts which have to be adapted to each system and instantiated when the requirement is reused	
*Requirement*: a statement that identifies a necessary attribute, capability, characteristic or quality of a system for it to have value and utility for an user	
*Requirements catalogue*: a set of generic and reusable requirements	
*Requirements traceability*: documentation of the relationships between requirements to facilitate the overall quality of the product under development, the understanding of the product and its artefacts, and the ability to manage change

**Table 6:** Questionnaire

**Q1**. Time devoted to the task (hours)
**Q2**. It is easy to reuse the requirements of a new project from the i18n requirements catalogue
**Q3**. The requirements of a new project can be quickly reused from the i18n requirements catalogue
**Q4**. The quality of the requirements obtained by reusing from the i18n requirements catalogue is high
**Q5**. The requirements obtained by reusing from the i18n requirements catalogue are understandable
**Q6**. Means of communication used (multiple options allowed)
Face-to-face meetings
Asynchronous (email)
Synchronous (chat, audio/video conference)
Other (please specify)
**Q7**. Problems encountered (if any)

## Abbreviations

CLM: co-located in Murcia
CLR: co-located in Rabat
CSS: cascading style sheets
GBL: games-based learning
GLO: global
GQM: goal/question/metric
GSD: global software development
GSDE: global software development education
MDI: Management Development Institute Gurgaon
MU: Marquette University
NL: natural language
PANGEA: Process for globAl requiremeNts enGinEering and quAlity
PNP: personal needs and preferences
RE: requirements engineering
SD: standard deviation
SE: software engineering
SQUARE: Security QUAlity Requirements Engineering
UL: University of Limerick
UMU: University of Murcia
UMV: Mohammed V University
XSLT: XSL transformations
